# Physician experiences and perceptions of patient-initiated recording in emergency departments: a multi-center survey in Southwestern China

**DOI:** 10.1186/s12910-026-01430-6

**Published:** 2026-04-01

**Authors:** Bo Jin, Lian-jing Liang, Chong-xi Xu, Ao Sha, Wei-kang Liu, Hao-ran Peng, Yang-chao Gan, Jiu-yu Gao, Zhi-hao Liu, Shao-yi Zhang, Dan Lu, Ya-rong He

**Affiliations:** 1 Department of Emergency Medicine, Institute of Disaster Medicine, Institute of Emergency Medicine, West China Hospital, Sichuan University, Chengdu, China; 2 Department of Otorhinolaryngology, Head & Neck Surgery, West China Hospital, Sichuan University, Chengdu, China; 3 Department of Otorhinolaryngology, Head & Neck Surgery, West China Tian Fu Hospital, Sichuan University, Chengdu, China; 4 Department of Neurosurgery, West China Hospital, Sichuan University, Chengdu, China; 5 The West China Second University Hospital, Sichuan University, Chengdu, China

**Keywords:** Patient-initiated recording, Covert recording, Emergency medicine, Physician-patient trust, Defensive medicine

## Abstract

**Background:**

With the widespread use of smartphones, patient-initiated recording in emergency departments (EDs) has become an increasing concern. In the high-pressure EDs environment, physicians frequently perceive such recordings as being motivated less by a desire to enhance understanding and more by a defensive intent to preserve evidence for potential disputes. This study investigates emergency physicians’ exposure to and perceptions of this phenomenon in Southwestern China.

**Methods:**

A cross-sectional survey was conducted via online snowball sampling among 306 emergency physicians from tertiary hospitals in Southwestern China between September and October 2025. The study assessed the frequency with which physicians encounter patient-initiated recording, associated clinical scenarios, response strategies, and perceptions of ethical and legal implications.

**Results:**

A total of 90.20% of respondents reported encountering patient-initiated recording within the past year. Among these physicians, 90.22% reported experiencing covert (undisclosed) recording, identifying the behavior through observation rather than patient disclosure. Commonly reported scenarios by the affected physicians included emergency diagnosis and treatment (77.90%) and dispute mediation (59.06%). Regarding attitudes, physicians rated perceived risks significantly higher than benefits on a 5-point Likert scale (1 = Strongly Disagree, 5 = Strongly Agree); while acknowledging moderate utility for elderly patients (mean score = 3.29), they indicated that recording was perceived to promote defensive medical behavior (mean score = 4.82) and induced psychological pressure (mean score = 4.74). Moreover, 72.55% believed the practice negatively impacted the physician-patient relationship. Furthermore, 90.20% expressed a strong demand for professional training, prioritizing case-based analysis of legal disputes (88.89%) and informed consent protocols (88.24%).

**Conclusions:**

The high reported exposure to patient-initiated recording—particularly in covert forms—among the surveyed emergency physicians in Southwestern China highlights physicians’ prevalent concerns about a perceived strain on trust and their self-reported association with defensive medical behaviors. Moving beyond strict prohibitions, healthcare institutions should consider prioritizing transparent governance and standardized communication protocols. Furthermore, targeted medico-legal training is essential to clarify rights and obligations, which could potentially help establish clearer institutional boundaries for managing patient-initiated recording.

**Trial registration:**

Not applicable (this study is a cross-sectional survey and does not involve any health care intervention).

**Supplementary Information:**

The online version contains supplementary material available at 10.1186/s12910-026-01430-6.

## Background

With the widespread adoption of smartphones, the phenomenon of patients initiating audio or video recordings during medical consultations has become increasingly common. According to surveys in Western countries, a significant proportion of patients (typically ranging from 15% to over 25%) have recorded their consultations, often to facilitate the review of medical advice, share information with family members, or enhance their involvement in decision-making [1–3]. Studies indicate that such recordings can improve information retention and treatment adherence, particularly in high-information load contexts such as oncology [4].

However, patient-initiated recording introduces complex psychological, ethical, and legal challenges. From the physician’s perspective, unacknowledged recordings may damage the trust in the physician-patient relationship and lead to defensive communication [5, 6]. Physicians often perceive such acts—especially when done without consent—as a form of surveillance that threatens their privacy [7, 8]. Regarding the legal landscape, diverse jurisdictions have different regulatory approaches to patient-initiated recording, with some jurisdictions emphasizing privacy protection and others placing more weight on patients’ access to information [9, 10].

Although patient recording behavior has been studied internationally, most evidence originates from Western countries [1, 2, 11]. Empirical data addressing the emergency medical environment in China remains limited, despite recent attention to professional attitudes [12]. Emergency departments (EDs) are characterized by rapid changes in patient conditions and high case complexity [7], often situated within a broader context of strained physician-patient trust and defensive medicine in China [13, 14]. Furthermore, there is a recognized legal tension between patients’ rights to information and physicians’ rights to privacy [9, 10]. This tension presents unique challenges within the legal framework in China (e.g., portrait rights). This unique environment makes physicians’ attitudes toward recording particularly significant. Currently, there is a lack of multi-center studies to guide management decisions. Therefore, this study aims to systematically analyze emergency physicians’ perceptions of patient-initiated recording, their responses, and their awareness of legal risks through a multi-center survey in Southwestern China. The findings aim to provide empirical support for optimizing physician-patient communication and developing management strategies.

## Materials and methods

This study employed a cross-sectional survey design to investigate the perceptions, attitudes, behavioral responses, legal risk awareness, and training needs of emergency physicians regarding patient-initiated recording of clinical encounters. The study was conducted from September to October 2025. Ethical approval was obtained from the Institutional Review Board of West China Hospital, Sichuan University (Approval No. 20251628).

The survey was anonymous and did not collect any directly identifiable personal information (e.g., names, ID numbers, or contact details). According to the ethical guidelines of the reviewing institution, the completion and submission of the questionnaire were considered to imply informed consent. All data were stored on password-protected servers and were accessible only to the core research team for the purpose of this study, ensuring strict data security and minimization.

### Participants

The survey was conducted among emergency physicians in Southwestern China using an online snowball sampling strategy via the ‘Questionnaire Star’ platform. Due to the anonymous design intended to encourage honest reporting on sensitive ethical issues, specific hospital names were not recorded. However, demographic data indicated that the vast majority of respondents (80.07%, *n* = 245) were employed at Tertiary A (Grade 3, Class A) hospitals, ensuring that the findings reflect the clinical reality of high-level regional medical centers. Inclusion criteria were defined as: (1) being a licensed physician currently working in an emergency department; and (2) willingness to participate in the study. Data exclusion criteria were established to ensure response quality. Questionnaires were considered invalid and excluded from analysis if they met the following conditions: (1) containing logical inconsistencies (e.g., discrepancies between reported age and years of practice); or (2) having a completion time of less than 120 s, suggesting a lack of engagement or seriousness in responding.

### Questionnaire design

The questionnaire was developed by the research team specifically for this study, drawing primarily on previous empirical studies and relevant guidelines on patient recording behaviors [1, 2]. The conceptual framework was grounded in prior literature [3, 5], addressing motivations, attitudes, and legal issues.

To ensure consistency, key operational definitions were explicitly provided to participants at the beginning of the survey:

•"Patient-initiated recording” was defined as any audio and/or video recording of the clinical encounter captured by patients or their companions using smartphones or other portable devices within the past 12 months.
•"Covert recording” was defined as recording activities initiated without the physician’s prior consent or notification, which were discovered by the physician solely through observation (e.g., noticing a raised phone) or third-party reports, rather than voluntary disclosure.

The draft questionnaire was reviewed by an expert panel consisting of three emergency physicians, two surgeons, and a medical law expert, each with at least five years of professional experience, to ensure clinical accuracy and legal validity. Based on their feedback, the items were refined. Subsequently, a pilot survey was conducted with 20 emergency physicians (excluded from the final sample) to further evaluate the clarity of the wording. The final instrument was structured into four domains: (1) Demographic and professional characteristics (e.g., age, gender, years of practice, hospital level); (2) Status of patient-initiated recording, covering prevalence, clinical scenarios, discovery modes, and physician response strategies; (3) Physician attitudes, assessed via a 5-point Likert scale measuring perceived risks (7 items) and benefits (10 items); and (4) Legal cognition and training needs, focusing on the admissibility of evidence and preferences for future medico-legal education. The full English version of the questionnaire is available in Supplementary File 1.

### Data collection

The questionnaire was distributed electronically via Wenjuanxing (www.wjx.cn), a widely used online survey platform in China. Each survey included a brief introduction explaining the study’s purpose and emphasizing voluntary participation. Distribution channels included academic conferences in emergency medicine, professional WeChat groups verified for medical personnel, and one-on-one outreach. The principal investigator oversaw the data collection process to ensure that respondents were verified members of the emergency medicine community.

### Statistical analysis

Data were exported from Wenjuanxing and validated by the principal investigator. Statistical analyses were performed using SPSS version 23 (IBM Corp., Armonk, NY, USA). Descriptive statistics were used to summarize the data. Continuous variables (e.g., age) were expressed as means with standard deviations (SD). Categorical variables (e.g., gender, professional title, and survey responses regarding recording scenarios) were presented as frequencies (n) and percentages (%). No missing data imputation was performed as all questions were mandatory in the electronic survey system.

## Results

### Demographic and professional characteristics

A total of 306 questionnaires were collected. All submissions passed the quality control checks (no logical inconsistencies and completion time ≥ 120 s). Consequently, no questionnaires were excluded, yielding a 100% validity rate among submitted questionnaires. While the use of online distribution precluded the calculation of a traditional response rate (total invited vs. participated), the high quality of submitted data suggests strong engagement among participants. The respondents represented a geographic distribution spanning multiple provinces, with the highest proportion of physicians practicing in Southwestern China. The study population was predominantly male (64.05%), with a mean age of 36.69 ± 7.05 years. The majority practiced in public (92.48%), tertiary A-level (80.07%), and teaching hospitals (76.14%). Regarding hospital types, most participants worked in general hospitals (92.48%), followed by maternal and child health hospitals (3.92%) and specialized hospitals (3.59%). Regarding the clinical environment, only 29.74% of respondents reported that their department was equipped with a dedicated communication room for recording or dispute mediation (Table 1). This facility is defined as a designated private space, often equipped with institutional audio-visual recording facilities, intended for documenting sensitive discussions and resolving disputes. The scarcity of such rooms indicates that the vast majority of clinical consultations still occur in open or semi-open environments, potentially fueling the patient’s desire to create their own record.

**Table 1:** Baseline characteristics of emergency physicians (*N* = 306)

Characteristic	Category	*n* (%)
Age (years)	≤ 30	76 (24.84)
	31–40	144 (47.06)
	41–50	76 (24.84)
	> 50	10 (3.27)
Gender	Male	196 (64.05)
	Female	110 (35.95)
Years in emergency medicine	≤ 5	85 (27.78)
	6–10	81 (26.47)
	11–20	103 (33.66)
	> 20	37 (12.09)
Professional title	Resident	99 (32.35)
	Attending	144 (47.06)
	Associate chief physician	45 (14.71)
	Chief physician	18 (5.88)
Highest degree	Bachelor or below	191 (62.42)
	Master	97 (31.70)
	Doctorate	18 (5.88)
Ownership type	Public	283 (92.48)
	Private	23 (7.52)
Hospital level	Tertiary A general hospital (top tier)	245 (80.07)
	Other levels	61 (19.93)
Hospital Type	General Hospital	283(92.48%)
	Maternal & Child Health	12(3.92%)
	Specialized Hospital	11(3.59%)
Teaching hospital	Yes	233 (76.14)
	No	73 (23.86)
Dedicated communication room	Yes	91 (29.74)
	No	215 (70.26)

Values are *n* (%). Percentages may not sum to 100% due to rounding

“Tertiary A general hospital” refers to the highest-tier general hospital in the mainland China classification (top-tier tertiary hospital)

Education Level: In the Chinese medical education system, the standard entry-level qualification for licensure is a 5-year undergraduate degree conferring a Bachelor of Medicine (BM). This degree renders graduates eligible for the National Medical Licensing Examination and allows them to practice as licensed physicians. This differs from systems requiring a postgraduate Doctor of Medicine (MD) degree

### Prevalence, clinical scenarios, and groups with higher propensity to record

**Fig. 1:**
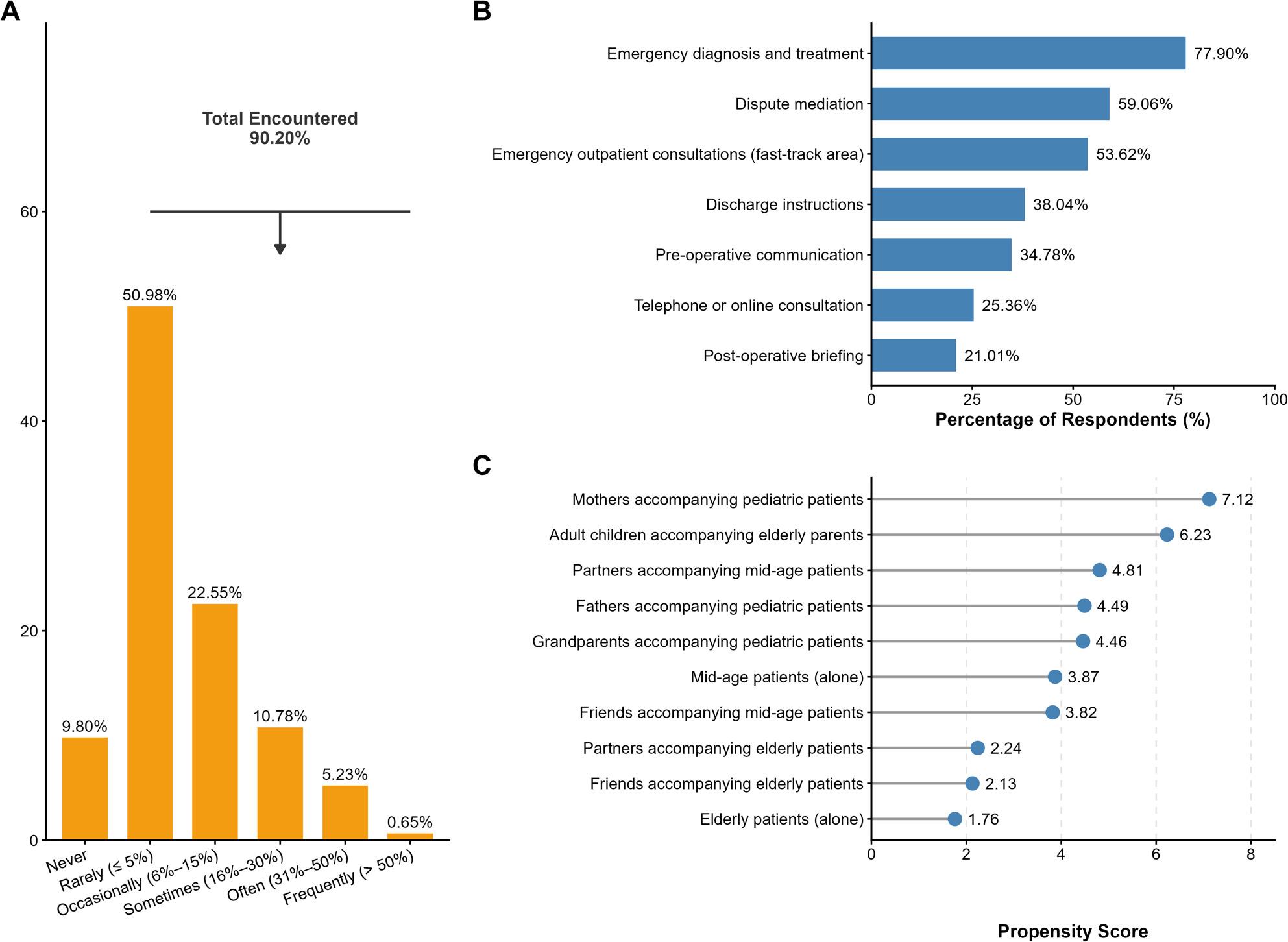
Prevalence and characteristics of patient-initiated recording in the Emergency Department. **A** Frequency of recording encounters reported by survey respondents (*n*=306) in the past year. The y-axis represents the percentage of physicians falling into each frequency category. The vast majority (90.20%) of physicians reported encountering patient recordings at least once. **B** Clinical scenarios where recordings occurred (*n*=276). Data represent the subset of respondents who reported encountering recordings. The most common scenarios were emergency diagnosis and treatment, followed by dispute mediation. **C** Physicians' perceptions of groups most likely to initiate recording. Data reflect the subjective rankings provided by respondents, not objective demographic statistics. Higher scores indicate a greater perceived likelihood of initiating recording. Intergenerational care scenarios (parents accompanying children, adult children accompanying elderly parents) were ranked as the most frequent drivers

The survey revealed that patient-initiated recording has become a common experience among the surveyed physicians. As illustrated in Fig. 1A, 90.20% of respondents reported encountering recording behavior at least once in the past year. Although the majority of these encounters were infrequent (50.98% reported a frequency of ≤ 5%), the high cumulative prevalence indicates that encountering recording has become a frequently reported aspect of practice for these respondents.

In terms of clinical context (Fig. 1B), recording encounters were most frequently reported in “conflict-prone” scenarios. Among physicians who encountered recording (*n* = 276), the vast majority reported that these incidents occurred during emergency diagnosis and treatment (77.90%, *n* = 215), followed by dispute mediation (59.06%, *n* = 163) and emergency outpatient consultations (fast-track area) (53.62%, *n* = 148). Fewer physicians reported encounters during standard procedural interactions such as discharge instructions (including rehabilitation guidance) (38.04%, *n* = 105).

Regarding the identity of the recorders, participants were asked to rank the groups they believed were most likely to initiate recording (Fig. 1C). In terms of perceived likelihood, physicians ranked mothers accompanying children as the group most prone to recording (composite score: 7.12), followed by adult children accompanying elderly parents (composite score: 6.23). In contrast, elderly patients presenting alone were perceived as the least likely group to record (composite score: 1.76). It is important to note that these figures reflect the physicians’ subjective observations of high-risk groups rather than objective demographic statistics of all recording events.

### Disclosure status, perceived impact, and response strategies

**Fig. 2:**
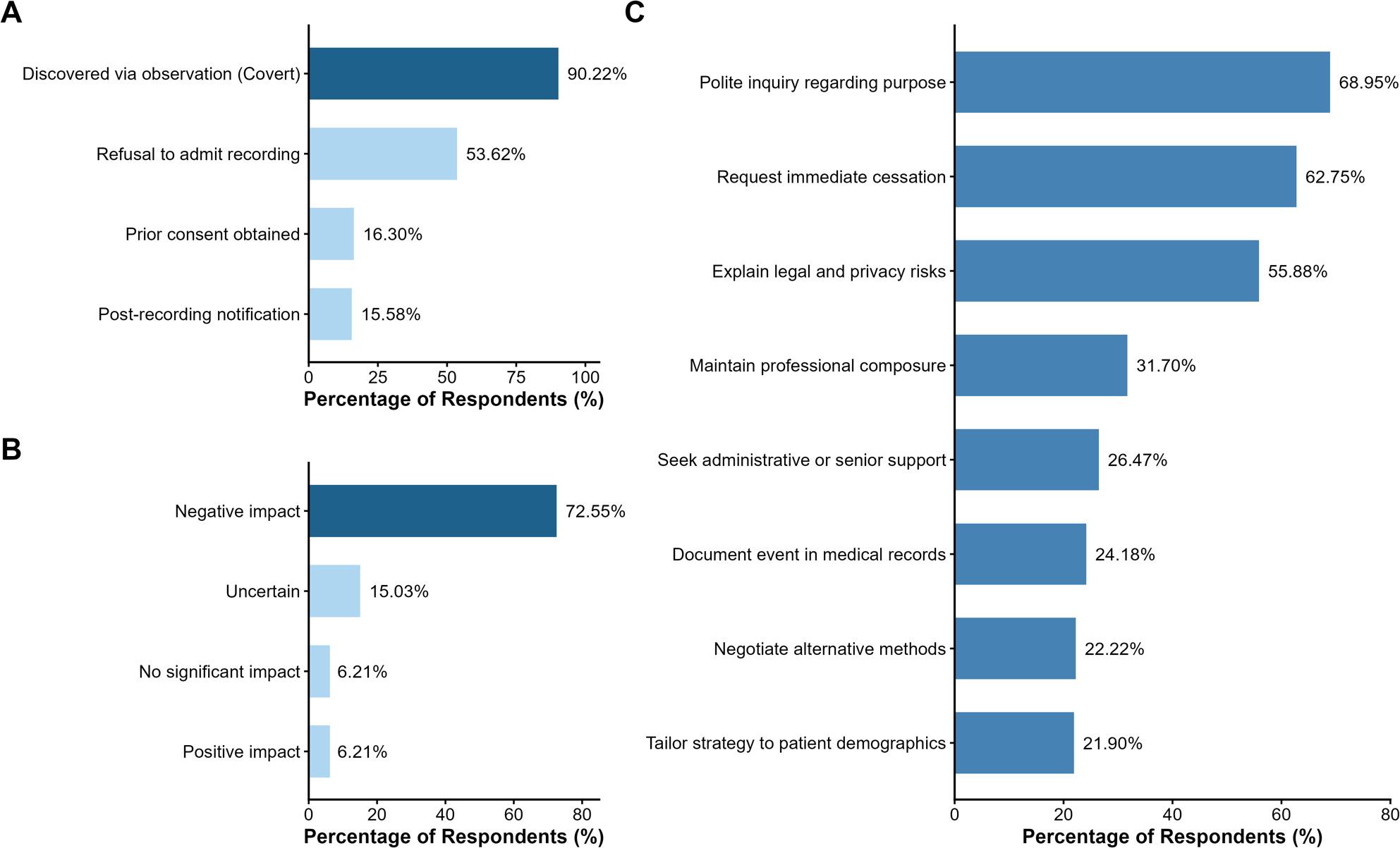
Disclosure status, impact, and response strategies. **A** Disclosure status. Analysis of the subset of physicians who encountered recording (*n*=276). 90.22% (*n*=249) of these respondents reported that the recording they encountered was covert (undisclosed). Only a minority reported obtaining prior consent or post-hoc disclosure. **B** Perceived impact. Physicians' sentiment regarding the impact of recording on the physician-patient relationship (*n*=306). **C** Response strategies. Actions taken by physicians when addressing potential or actual recordings

Analysis of recording encounters (Fig. 2A) reveals that the practice is predominantly covert. Among the subgroup of respondents who reported encountering recordings (*n* = 276), 90.22% (*n* = 249) discovered the behavior via observation rather than patient disclosure. Regarding consent, only 16.30% (*n* = 45) of these physicians reported instances where prior consent was obtained, while 15.58% (*n* = 43) reported post-hoc disclosure. Notably, more than half of these physicians (53.62%, *n* = 148) reported instances where patients refused to admit to recording even when directly asked.

Regarding the perceived impact on the therapeutic alliance (Fig. 2B), physician sentiment across the entire cohort (*n* = 306) was predominantly negative. The majority of participants (72.55%) believed that patient-initiated recording exerts a negative impact on the physician-patient relationship. In contrast, only 6.21% perceived a positive impact.

In terms of response strategies (Fig. 2C), physicians (*n* = 306) reported employing a mix of communicative and defensive measures when addressing potential or actual recordings. The most common immediate response was to politely inquire about the purpose of the recording (68.95%), followed closely by requesting immediate cessation (62.75%) and explaining legal and privacy risks (55.88%).

### Physician attitudes: a risk-benefit analysis

**Fig. 3:**
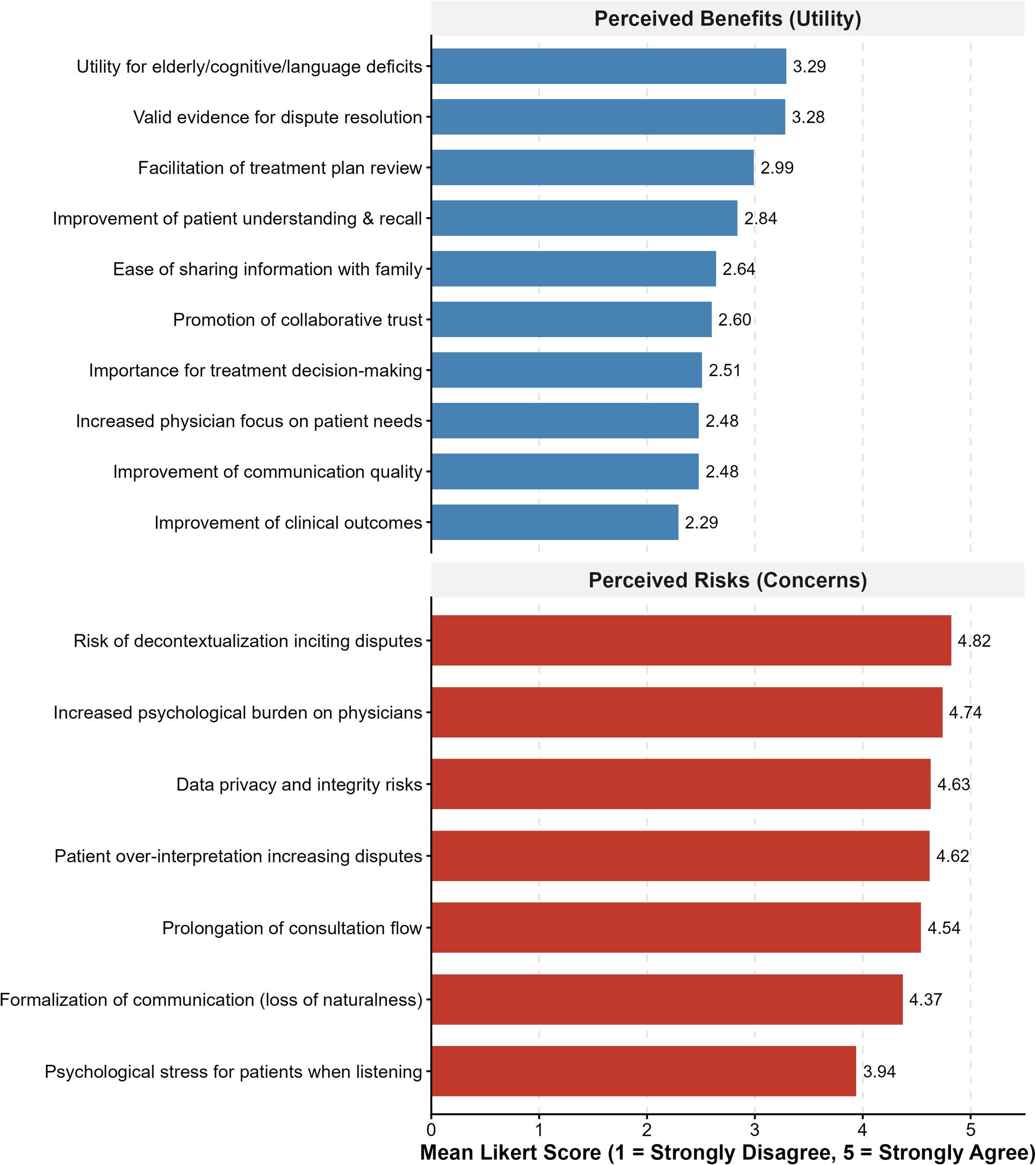
Physician attitudes toward the risks and benefits of patient-initiated recording. Data represent the mean scores on a 5-point Likert scale (1 = Strongly Disagree, 5 = Strongly Agree). The chart is stratified into two panels: Perceived Risks (red bars) and Perceived Benefits (blue bars). Physicians consistently rated risk-related items significantly higher (Mean > 4.0) than benefit-related items. The primary concerns were decontextualization (Mean = 4.82) and psychological pressure (Mean = 4.74), whereas perceived utility was generally low (Mean < 3.3)

As shown in Fig. 3, the survey revealed a significant asymmetry in physicians’ attitudes toward the practice. Among the 17 items assessed, physicians consistently rated perceived risks significantly higher than potential benefits.

Regarding risks (Fig. 3, Top Panel), participants expressed strong agreement with all seven negative statements. The primary concern was the potential for decontextualization, where recordings might be used out of context to incite disputes (Mean = 4.82). This was closely followed by concerns that recording increases physician psychological burden (Mean = 4.74) and poses risks to data privacy and integrity (Mean = 4.63).

Conversely, physicians were skeptical of the benefits (Fig. 3, Bottom Panel). While there was moderate neutrality regarding the utility of recording for elderly or cognitively impaired patients (Mean = 3.29) and as valid evidence for dispute resolution (Mean = 3.28), the majority of benefit-related items received negative ratings (Mean < 3.0). This significant disparity suggests that emergency physicians perceive the practice as carrying high professional liability with minimal utility, reflecting a prevalent defensive stance among the respondents.

### Legal cognition and specific knowledge gaps

Although the vast majority of emergency physicians have encountered patient recording, their self-assessment of relevant legal knowledge was notably low (Mean = 3.17 on a 0–10 scale). However, when asked about specific legal issues involved in recording, physicians demonstrated high awareness of the physician’s right to consent (88.24%), physician’s right to know (85.62%), and patient privacy protection (85.29%).

Physicians’ judgment on the admissibility of recordings as evidence revealed a nuanced understanding of legal distinctions (Table 2). The vast majority (> 80%) correctly identified that recordings obtained through open consent (88.24%) or administrative mediation (85.29%) are likely admissible. Conversely, 86.60% believed that covert recordings in private settings (e.g., a physician’s home) should be inadmissible. However, there was notable uncertainty regarding covert recordings made within the consulting room, with 25.82% believing they might be valid, highlighting a critical area for legal education.

**Table 2:** Emergency physicians’ judgment on the admissibility of recordings as legal evidence (*n* = 306)

Scenario	Admissible(Yes)	Inadmissible(No)
High Consensus: Likely Admissible		
Open consent: Recording with explicit notification and physician’s verbal consent	270 (88.24%)	36 (11.76%)
Authenticity: Untampered recording reflecting key clinical exchanges	267 (87.25%)	39 (12.75%)
Formal mediation: Agreement recorded during a formal mediation session	261 (85.29%)	45 (14.71%)
Corroboration: Supplementary evidence corroborating medical records	252 (82.35%)	54 (17.65%)
High Consensus: Likely Inadmissible		
Covert recording in private spaces (e.g., home, car, locker room)	41 (13.40%)	265 (86.60%)
Illegal means: Recording obtained via coercion, inducement, or fraud	43 (14.05%)	263 (85.95%)
Low Consensus / Controversial		
Bedside recording: Recorded by family members at the bedside	117 (38.24%)	189 (61.76%)
Physician’s covert recording: Recorded by physician without patient notification	79 (25.82%)	227 (74.18%)

(Data are presented as number (percentage). Scenarios are categorized based on the consensus level of emergency physicians’ responses)

**Fig. 4:**
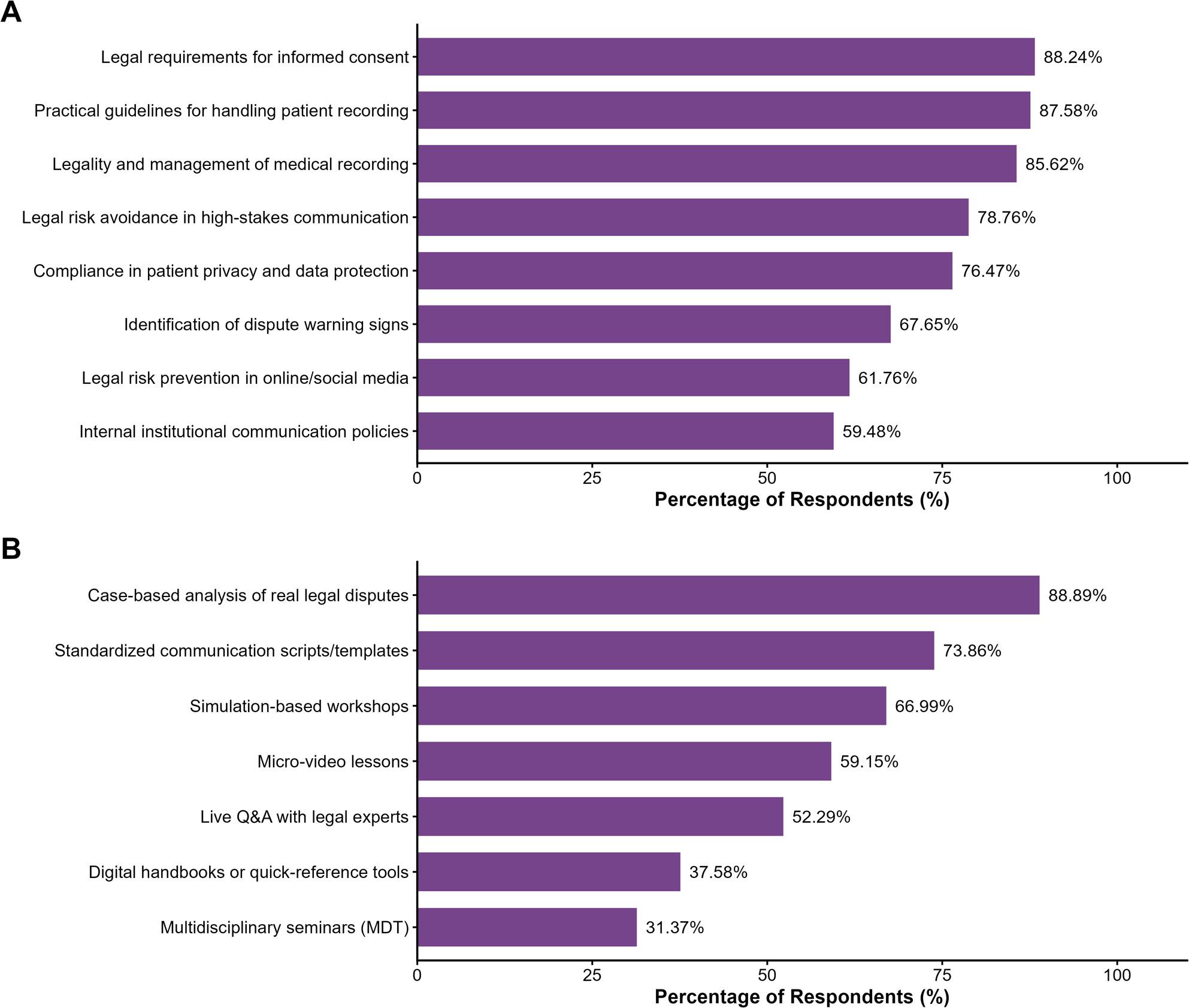
Legal knowledge needs and preferred training formats among emergency physicians. **A** Priority topics for legal and communication training (*n*=306). The most requested topics were legal requirements for informed consent (88.24%) and practical guidelines for handling patient recording (87.58%). **B** Preferred formats for training delivery (*n*=306). Physicians favored practice-based formats, with "Case-based analysis of real legal disputes" (88.89%) being the top choice. (Note: Data are presented as percentages of respondents selecting each option. The unified purple color scheme represents the proposed educational interventions. Responses classified as 'Other' [<2%] were excluded for visual clarity.)

Given these challenges, there was a significant consensus (90.20%) regarding the need for communication skills improvement. Figure 4 illustrates the specific legal knowledge needs and preferred training formats. Regarding training topics (Fig. 4A), the most requested areas were the legal requirements for informed consent (88.24%) and practical guidelines for handling patient recording (87.58%). Regarding training delivery (Fig. 4B), emergency physicians demonstrated a strong preference for practice-based learning, with “Case-based analysis of real legal disputes” (88.89%) being the top choice, followed by standardized communication scripts (73.86%).

## Discussion

### Normalization of covert recording and perceived challenges to trust

The findings of this multi-center survey reveal a notable finding: patient-initiated recording was reported as a highly prevalent experience among the surveyed emergency physicians. We found that 90.20% of emergency physicians had encountered recording behavior in the past year. It is crucial to distinguish this “physician exposure rate” from patient-reported prevalence. While studies in Western primary care and outpatient settings assessing patient-reported behaviors indicate that approximately 15%–20% of patients report recording their consultations [1–3], our data measures the cumulative probability of an ED physician encountering this behavior over time. Our high exposure rate (90.20%) aligns with recent literature noting that over 70% of medical professionals in China have experienced being recorded [12]. This consistency suggests that within this surveyed cohort in Southwestern China, exposure to recording was commonly reported among these participating frontline clinicians. However, the *nature* of these encounters in the ED appears distinct. Oyedokun et al. (2019) highlighted that in the emergency setting, there is a fundamental disconnect: while 62% of patients wish to record, only 28% of clinicians feel comfortable permitting it [7]. This conflict, as perceived by the physicians in our study, may be exacerbated in the Chinese context by what has been described in the literature as “presupposed distrust”—a phenomenon characterized by Li et al. (2022) as pre-existing skepticism rooted in social factors, where patients are posited to view medical interactions through a lens of suspicion [13]. Consequently, unlike US oncologists, the majority of whom report that patients ‘usually’ or ‘always’ ask permission before recording [15], our study found that among ED physicians who had experienced recording, 90.22% reported it as a covert event. This stark contrast—even when compared to the 40% covert rate cited in the context of UK general practice [11]—suggests that in our sample, physicians predominantly reported perceiving smartphones as tools for ‘defensive evidence’ gathering rather than utilized as memory aids [5].

### Risk-benefit asymmetry and the “Panopticon” effect

Our results highlight a distinct asymmetry in physicians’ attitudes: the perceived professional risks overwhelmingly outweigh the clinical benefits. Physicians expressed strong concerns regarding decontextualization and psychological pressure. This reflects what Ryan et al. (2022) describe as feeling “disrespected” and experiencing a loss of control [6]. In the high-pressure ecosystem of the ED, this pattern may be interpreted through what has been described in the literature as a “Panopticon effect”—the feeling of being constantly observed. As argued by Buckman (2015), this sense of surveillance is reported to induce “defensive medicine,” potentially prompting physicians to adopt formalized, cautious communication rather than engaging in empathetic dialogue [8]. Although this study did not directly assess experiences of workplace violence, our data suggests this defensive stance may be closely associated with such broader fears. Wu et al. (2025) recently identified “unmet patient expectations” as a primary driver of aggression against Chinese physicians [14], explaining why an unconsented camera may be perceived not just as a privacy breach, but as a potential precursor to conflict. Interestingly, this fear of legal liability may be disproportionate to the actual risk. A recent study by Naeem et al. (2024) found no statistical increase in malpractice claims against physicians who utilized video recordings of clinic visits [16]. This suggests that the physicians’ anxiety is driven more by the *uncertainty* of the recording’s intent than by empirical legal outcomes.

### Legal complexity and the imperative for active governance

This study reveals a critical gap between the high reported exposure to recording and the lack of regulatory clarity. As Schwartz (2023) argues, current privacy frameworks are often a “poor fit” for the clinical context [9]. In the Chinese context, this complexity is exacerbated by the tension within the Civil Code, which protects a physician’s “portrait rights” yet grants patients broad rights to access their medical information. This legal ambiguity leaves emergency physicians in a precarious position, which they report as fueling their anxiety and defensive behaviors. Our findings, aligned with physicians’ reported experiences, suggest that simply attempting to prohibit recording may be insufficient and may not alleviate the underlying erosion of trust. Ryan et al. (2022) found that when physicians refuse recording, it often triggers the patient’s suspicion (“What are you hiding?“), which can undermine the consumer-clinician relationship [17]. Instead, healthcare institutions should consider shifting from prohibition to transparency. Evidence from the “OpenNotes” movement (DesRoches et al., 2020) demonstrates that while clinicians initially fear that sharing medical records will increase workload and liability, actual implementation often leads to improved trust without the feared negative consequences [18]. Similarly, the concept of open recording advocated by Elwyn et al. (2015) offers a practical template for adoption [8]. Institutions implementing this model encourage patients to ask for permission (“Can I record?“) and train staff to agree, thereby validating the patient’s need for information retention while establishing clear boundaries regarding the privacy of third parties. To support this shift, we recommend a targeted educational strategy that moves beyond abstract legal theory to practical application. Training should clarify that utilizing recordings rarely increases malpractice liability and can exonerate physicians providing standard care [16], while also equipping staff with specific de-escalation scripts to handle requests confidently rather than defensively. Therefore, by adopting an open and collaborative approach—as suggested by Rimmer (2019)—that encourages patients to record openly with consent [19], and normalizing recording as a legitimate tool for care coordination [4], the “covert” nature of the act can be de-escalated, protecting both patient rights and professional dignity.

### Study limitations

Several limitations should be considered when interpreting these findings. First, as a cross-sectional survey, this study captures a snapshot of attitudes and experiences but cannot establish causality between recording behaviors and the deterioration of the physician-patient relationship. Second, the sample was primarily drawn from tertiary A-level hospitals in Southwestern China. While this region is representative of the high-volume, high-acuity medical centers in China, the findings may not be fully generalizable to primary care clinics or other geographic regions with different resource distributions. Third, this study relies exclusively on self-reported data from physicians. We did not survey patients, meaning our data on “recording motivations” reflects physicians’ perceptions rather than patients’ actual intent. It is possible that physicians, operating in a defensive mindset, may misinterpret benign recording behaviors (e.g., for memory aid) as hostile evidence-gathering. Fourth, interpretations of the data must account for potential biases inherent in the study design. Given the retrospective nature of the survey (covering the past year), findings are subject to recall bias; physicians may be more likely to remember and report salient, conflict-ridden encounters involving recording, potentially overestimating the prevalence of negative outcomes compared to neutral recording events. Additionally, given the voluntary nature of the survey, selection bias cannot be ruled out. Physicians who feel more negatively about recording or who have experienced recent conflicts may have been more motivated to participate than those with neutral experiences. Finally, given the “covert” nature of the recordings, it is possible that some recordings went undetected; therefore, the reported exposure rate reflects only physicians’ recognized encounters. Future research should employ matched physician-patient surveys or observational designs to bridge the gap between perceived threats and actual patient behaviors.

## Conclusions

In this multi-center survey of emergency physicians in Southwestern China, patient-initiated recording—predominantly in covert form—emerged as a common experience among the surveyed physicians rather than an exceptional event. Physicians perceived such recording as strongly risk-oriented, reporting high levels of concern about decontextualization, privacy breaches, and psychological burden, with minimal perceived utility. Covert recording was also associated, in physicians’ reports, with self-reported increases in defensive medical behaviors and a perceived strain on the physician-patient relationship. Furthermore, respondents demonstrated substantial uncertainty regarding the legal status of different recording scenarios and expressed a strong demand for practical, case-based training in communication and medico-legal risk management.

Given these findings, healthcare institutions may consider whether developing clear and practicable protocols that define when and how patient-initiated recording is acceptable could encourage patients to disclose their intention to record, and support physicians in responding consistently. We suggest that transparent, mutually agreed recording protocols could represent a pragmatic step toward reframing “hidden surveillance” into “shared documentation.” Such an approach may help support communication, potentially mitigate self-reported defensive practices, and foster what might be an environment more conducive to rebuilding trust in high-pressure emergency settings.

## Supplementary Information

Supplementary Material 1.

## Data Availability

The datasets used and/or analysed during the current study are available from the corresponding author on reasonable request.

## Abbreviations

EDs: Emergency Departments
SD: Standard Deviation
