# Supplementary material for: Physician experiences and perceptions of patient-initiated recording in emergency departments: a multi-center survey in Southwestern China
Source: BMC Med Ethics. 2026 Apr 1;27:91. doi: 10.1186/s12910-026-01430-6 (PMC13169550; doi:10.1186/s12910-026-01430-6)
Supplement: Supplementary file 1 — Supplementary Material 1. [file 12910_2026_1430_MOESM1_ESM.pdf]

Supplementary File 1: Survey Questionnaire

Title: Survey on the Cognition of Patient-Initiated Recording and Training Needs for Legal Risk Prevention among Emergency Physicians

Instructions: *Dear Colleague, We are conducting a study to understand the current status of patient-initiated recording in the Emergency Department (ED). This survey is anonymous. Please answer based on your actual experience. The data will be used for academic research purposes only. Thank you for your participation.*

Informed Consent: Submission of this questionnaire implies your consent to participate. ○ Yes, I agree to participate. ○ No (End of Survey)

Part 1: Demographic and Professional Characteristics

Q1. Your age: ________ years old

Q2. Your gender:

○ Male

○ Female

Q3. Years of practice in emergency medicine: ________ years

Q4. What is the level of your hospital?

○ Tertiary A (Grade III, Level A)

○ Tertiary B (Grade III, Level B)

○ Secondary A (Grade II, Level A)

○ Secondary B (Grade II, Level B) or below

Q5. Is your hospital a teaching hospital?

○ Yes

○ No

Q6. What is the ownership type of your hospital?

○ Public hospital

○ Private hospital

Q7. What is the type of your hospital?

○ General Hospital

○ Maternal and Child Health Hospital / Women and Children's Hospital

○ Specialized Hospital

Q8. Your highest educational degree:

○ Junior College or below

○ Bachelor’s degree

○ Master’s degree

○ Doctoral degree (Ph.D./M.D.)

Q9. Your professional title:

○ Resident

○ Attending Physician

○ Associate Chief Physician

○ Chief Physician

Q10. Is your department equipped with a dedicated communication room (for recording or dispute mediation)?

○ Yes

○ No

Part 2: Status of Patient-Initiated Recording

Q11. In the past year, how often have you encountered patients or their families recording (audio or video) during your clinical practice?

○ Never

○ Rarely (≤ 5%)

○ Occasionally (6%–15%)

○ Sometimes (16%–30%)

○ Often (31%–50%)

○ Frequently (> 50%)

Q12. In which clinical scenarios have you encountered patient recording? (Multiple Choice)

□ Outpatient consultation (e.g., initial visit, follow-up)

□ Pre-operative communication (e.g., surgical plan, risk disclosure)

□ Post-operative briefing (e.g., surgical outcomes, complications)

□ Discharge instructions (e.g., medication guidance, rehabilitation plans)

□ Emergency diagnosis and treatment (e.g., rapid communication in urgent situations)

□ Telephone or online consultation (e.g., telemedicine, phone follow-up)

□ Dispute mediation (e.g., discussing controversial issues)

Q13. Based on your clinical experience, please rank the following patient groups

by their propensity to initiate recording (1 = Highest propensity). [Please select at least 5 items and rank them]

□ Mothers accompanying pediatric patients

□ Adult children accompanying elderly parents

□ Partners accompanying young/middle-aged patients

□ Fathers accompanying pediatric patients

□ Grandparents accompanying pediatric patients

□ Young/middle-aged patients (alone)

□ Friends accompanying young/middle-aged patients

□ Partners accompanying elderly patients

□ Friends accompanying elderly patients

□ Elderly patients (alone)

Q14. What were the characteristics of the recording behaviors you encountered? (Multiple Choice)

□ Discovered via observation (Covert recording)

□ Refusal to admit recording

□ Prior consent obtained

□ Post-recording notification

□ Other (Please specify: __________)

Q15. In your clinical practice, how acceptable do you consider patient-initiated recording of the medical encounter? [Please rate on a scale of 0 to 10]

○ 0 (Not acceptable at all)

○ 1

○ 2

○ 3

○ 4

○ 5

○ 6

○ 7

○ 8

○ 9

○ 10 (Perfectly acceptable)

Q16. What impact do you believe patient-initiated recording has on the physician-patient relationship?

○ Positive impact

○ Negative impact

○ No significant impact

○ Uncertain

Q17. When you discover that a patient is recording the medical encounter without prior notification, what measures do you take? [Multiple Choice]

□ Maintain professional composure and continue the consultation (Do not interrupt the patient; ensure the patient's condition is properly handled)

□ Politely inquire about the purpose of the recording (Understand their true intent in a non-confrontational manner)

□ Explain legal and privacy risks (Inform the patient about potential legal issues such as privacy protection and evidence admissibility)

□ Request immediate cessation of the recording (Explain reasons such as privacy protection or avoiding misunderstanding)

□ Document the event in the medical record (Record the time, location, and device used for future reference)

□ Seek support from senior physicians or hospital administration (If the situation is complex or cannot be handled properly)

□ Negotiate alternative methods (Suggest stopping the recording and using other ways, such as written notes, to record the process)

□ Tailor strategy based on patient demographics (e.g., communicate with parents for pediatric patients; provide detailed explanations for elderly patients with hearing/cognitive impairments)

□ Other (Please specify: __________)

Part 3: Physician Attitudes (Risk-Benefit Analysis)

Q18. The following statements relate to your views on patient-initiated recording. Please indicate your level of agreement with each statement based on your current feelings. There are no right or wrong answers. Please respond intuitively. [Matrix Scale Question] (Scale: 1 = Strongly Disagree, 2 = Disagree, 3 = Neutral/Uncertain, 4 = Agree, 5 = Strongly Agree)

1. Risk of decontextualization: Recording content may be taken out of context,

potentially inciting doctor-patient disputes or legal issues.

○ 1 ○ 2 ○ 3 ○ 4 ○ 5

2. Psychological burden: Recording increases the psychological pressure and burden on physicians.

   ○ 1 ○ 2 ○ 3 ○ 4 ○ 5

3. Data privacy risks: Loopholes in data management may lead to personal information leakage or risks to data integrity (e.g., tampering).

   ○ 1 ○ 2 ○ 3 ○ 4 ○ 5

4. Over-interpretation: Patients or their families may over-interpret the recorded content, increasing the risk of disputes.

   ○ 1 ○ 2 ○ 3 ○ 4 ○ 5

5. Prolongation of consultation: Recording extends the duration of the consultation and disrupts the workflow.

   ○ 1 ○ 2 ○ 3 ○ 4 ○ 5

6. Formalization of communication: Recording leads to formalized communication, reducing the naturalness of the medical interaction.

   ○ 1 ○ 2 ○ 3 ○ 4 ○ 5

7. Patient stress: Listening to the recording afterwards may cause psychological stress for the patient or their family.

   ○ 1 ○ 2 ○ 3 ○ 4 ○ 5

8. Utility for vulnerable populations: Recording has a positive effect for elderly patients or those with language/cognitive impairments.

○ 1 ○ 2 ○ 3 ○ 4 ○ 5

9. Evidence for dispute resolution: Recording serves as valid evidence to protect rights and build a mechanism for resolving medical disputes.

   ○ 1 ○ 2 ○ 3 ○ 4 ○ 5

10. Review of treatment plans: Recording allows patients/families to better review medical information (e.g., treatment plans, medication instructions).

    ○ 1 ○ 2 ○ 3 ○ 4 ○ 5

11. Understanding and recall: Recording improves patients' and families' understanding and retention of medical information.

    ○ 1 ○ 2 ○ 3 ○ 4 ○ 5

12. Information sharing: Recording facilitates the sharing of medical plans with family members or other healthcare providers.

    ○ 1 ○ 2 ○ 3 ○ 4 ○ 5

13. Collaborative relationship: Recording promotes an equal, collaborative, and trusting relationship between doctors and patients.

    ○ 1 ○ 2 ○ 3 ○ 4 ○ 5

14. Decision-making: Recording is significant for the treatment decision-making process.

    ○ 1 ○ 2 ○ 3 ○ 4 ○ 5

15. Physician focus: Recording enables physicians to focus more on the needs of the patient and their family.

    ○ 1 ○ 2 ○ 3 ○ 4 ○ 5

16. Communication quality: Recording improves the quality of doctor-patient communication.

○ 1 ○ 2 ○ 3 ○ 4 ○ 5

17. Clinical outcomes: Recording can improve the patient's treatment outcomes.

○ 1 ○ 2 ○ 3 ○ 4 ○ 5

Part 4: Legal Cognition and Training Needs

Q19. How would you rate your knowledge regarding the laws and regulations related to medical recording in China? [Self-assessment Scale] (Scale: 0 = Know nothing at all, 10 = Know very well)

○ 0

○ 1

○ 2

○ 3

○ 4

○ 5

○ 6

○ 7

○ 8

○ 9

○ 10

Q20. What legal issues do you think are involved in patient-initiated recording?

[Multiple Choice]

□ Physician's right to consent

□ Physician's right to know

□ Protection of patient privacy

□ Determination of medical disputes

□ Legality/Admissibility of evidence

□ Personal information and data security/storage

□ Other

Q21. In your opinion, under which of the following scenarios can a recording be used as legally valid and admissible evidence? [Matrix Scale Question] (Options: Yes / No)

1. Open consent: The patient explicitly notifies you and obtains your verbal consent to record the explanation of surgical risks in the consulting room.
2. Authenticity: The recording clearly and completely captures key clinical exchanges (e.g., informed consent) without tampering, accurately reflecting the situation.
3. Corroboration: The recording serves as supplementary evidence that corroborates other objective evidence, such as medical records and test reports, forming a complete evidentiary chain.
4. Formal mediation: A recording of an agreement on compensation reached during a formal mediation session where both parties were present and aware.
5. Covert recording in private spaces: A secret recording of the physician's private

remarks (e.g., complaints) made in the physician's home, private car, or locker room without their knowledge or consent.

6. Physician's covert recording: A complete and authentic recording of the dialogue made by the physician during a consultation without notifying the patient.
7. Illegal means: A recording obtained through coercion, inducement, fraud, or other illegal means.
8. Bedside recording: A recording made by family members at the bedside capturing the physician-patient communication regarding the patient's condition.

Q22. In your opinion, do emergency physicians need to improve their communication skills regarding medical recording?

○ Yes

○ No

○ Uncertain

Q23. Which specific areas of legal knowledge regarding doctor-patient communication do you wish to learn? [Multiple Choice]

□ Legal requirements for informed consent (Statutory requirements for risk disclosure and documentation)

□ Practical guidelines for handling patient recording (Standard procedures when encountering recording)

□ Compliance in patient privacy and personal information protection

□ Legality and management of medical recording (Validity of evidence and storage rules)

□ Legal risk avoidance strategies in high-stakes communication

□ Legal risk prevention in online/social media consultations

□ Identification of dispute warning signs and compliant response protocols

□ Internal institutional communication management policies

□ Other

Q24. What are your preferred formats for learning knowledge related to medical recording? [Multiple Choice]

□ Case-based analysis of real legal disputes (Analysis of court rulings on actual disputes)

□ Standardized communication scripts/templates training

□ Simulation-based workshops (Scenario simulation teaching)

□ Micro-video lessons

□ Live Q&A with legal experts (Online live broadcast)

□ Digital handbooks or quick-reference tools

□ Multidisciplinary seminars (MDT)

□ Other
